# Combined tonsillar reflex zone stimulation and four-step manipulative reduction for Grisel syndrome following pediatric adenotonsillectomy: a case report

**DOI:** 10.3389/fped.2026.1744987

**Published:** 2026-06-03

**Authors:** Yiding Zhao, Ruqi Zhang, Qiang Wang

**Affiliations:** 1 College of Acupuncture, Moxibustion and Tuina, Shandong University of Traditional Chinese Medicine, Jinan, China; 2 Department of Tuina, Shandong Provincial Hospital Affiliated to Shandong First Medical University, Jinan, China

**Keywords:** case report, four-step manipulative reduction, Grisel syndrome following adenotonsillectomy, manual stimulation of the tonsillar reflex zone, tuina

## Abstract

**Background:**

Grisel syndrome (GS), also referred to as nontraumatic atlantoaxial subluxation, is typically associated with head and neck infections, inflammation, or postoperative complications. Its clinical manifestations include torticollis, neck pain and stiffness, restricted cervical mobility, and muscle spasms. Based on the pathological features of GS following tonsillectomy and adenoidectomy, as well as the anatomical relationship between the palatine tonsils and the atlantoaxial joint, this report proposes a therapeutic approach combining manual stimulation of the tonsillar reflex zone with a four-step atlantoaxial manipulative reduction for the management of this condition.

**Methods:**

A 7-year-old Chinese boy presented with neck pain, cervical deviation, and restricted range of motion persisting for over two weeks. The patient demonstrated marked limitation in left cervical rotation, with mild restrictions in right rotation, flexion, and extension. Based on the patient's clinical presentation, the primary therapeutic intervention consisted of manual stimulation of the tonsillar reflex zone combined with an atlantoaxial manipulative reduction protocol.

**Results:**

After nine days of treatment, the patient demonstrated resolution of cervical pain with improvement in cervical flexion, extension, and rotational movements. Tonsillar region discomfort had also significantly resolved. At the 15-day follow-up assessment, both cervical alignment and functional mobility were restored to normal physiological parameters, with no recurrence of tonsillar or cervical pain reported.

**Conclusion:**

This clinical case demonstrates that manual stimulation of the tonsillar region can alleviate local inflammation, while combined with cervical soft tissue relaxation and atlas-axis joint reduction to restore normal anatomical alignment. This integrated protocol successfully achieved therapeutic goals for this condition.

## 1 Introduction

Atlantoaxial subluxation refers to a condition where the atlas or axis vertebra displaces posteriorly, anteriorly, or laterally, or undergoes relative rotation, leading to an altered physiological relationship between the atlas and the odontoid process. This displacement cannot self-reduce, resulting in instability of the intervertebral structures and subsequent irritation or compression of surrounding soft tissues such as muscles, nerves, ligaments, and blood vessels (1). Current evidence suggests that the etiology and related mechanisms of atlantoaxial subluxation involve the combined anatomical, biomechanical, and histological characteristics of the atlantoaxial joint and its surrounding structures (2). Grisel syndrome (GS), also known as non-traumatic atlantoaxial subluxation, was first described by Grisel. It is commonly associated with infections or inflammatory processes in the head and neck region (such as tonsillitis or nasopharyngitis) or following head and neck surgeries (e.g., tonsillectomy and adenoidectomy). Clinical manifestations include torticollis, neck pain and stiffness, restricted neck movement, muscle spasms, dysphagia, fever, and hearing loss. In severe cases, neurological symptoms may occur due to spinal cord or nerve root compression, leading to arm numbness, weakness, paresthesia, or gait instability.

Current standard treatments for GS include conservative management and surgical intervention. Conservative approaches primarily involve cervical immobilization using a collar or brace, traction, and pharmacotherapy. Traction is typically applied via an occipitomandibular harness. In severe cases, cranial traction may be employed for reduction. Pharmacological treatment consists of nonsteroidal anti-inflammatory drugs, muscle relaxants, and analgesics to control pain and inflammation. Additionally, given the frequent occurrence of infection following tonsillectomy, antimicrobial therapy is often indicated. For patients who do not respond to conservative measures, surgical options such as atlantoaxial fusion, atlantoaxial release, or decompressive reconstruction of the atlantoaxial joint are commonly performed. However, the commonly used treatment modalities have several limitations. Conservative treatment is associated with a prolonged therapeutic course and suboptimal efficacy. Surgical intervention carries significant risks and high costs, and it may be poorly accepted by patients and their families. Multiple studies have indicated that alleviating local inflammatory swelling and infiltration at the surgical site is crucial for treating postoperative GS. Both conservative and surgical approaches have certain shortcomings in this regard. Therefore, it is particularly important to seek a safe, effective, and well-accepted clinical treatment for this condition.

This report describes the case of a patient with GS following adenotonsillectomy who was treated with combined manual stimulation of the tonsillar reflex zone and a four-step manipulative reduction. After treatment, the patient exhibited improvement in cervical mobility and marked reduction of para-atlantoaxial tenderness. The combined therapeutic approach maintained a stable clinical efficacy without recurrence of symptoms.

## 2 Case presentation

### 2.1 Medical history materials

A 7-year-old male patient presented with a 6-month history of snoring during sleep accompanied by abnormal breathing of unknown cause. His symptoms were characterized by nocturnal snoring, mouth breathing, occasional apnea, and sleep interruptions due to a sensation of choking. The child also experienced intermittent nasal congestion. There was no significant rhinorrhea, tinnitus, hearing loss, dizziness, or headache. He had no recent history of fever, cough, or sputum production. While he occasionally exhibited retching, no obvious nausea was reported. For further evaluation and management, the patient was admitted to Shandong Provincial Hospital Affiliated to Shandong First Medical University. He was diagnosed with tonsillar hypertrophy accompanied by adenoid hypertrophy in the outpatient clinic and scheduled for surgical intervention. Physical examination revealed no significant mucosal hyperemia, but bilateral tonsils were enlarged to grade III. No other obvious masses were identified. Flexible fiberoptic laryngoscopy confirmed adenoid and tonsillar hypertrophy. Preoperative diagnosis was tonsillar hypertrophy with adenoid hypertrophy. On January 30, 2025, the patient underwent surgery, during which bilateral tonsillar hypertrophy and adenoid tissue obstructing the posterior choanae were confirmed. Total tonsillectomy and adenoidectomy were performed. Postoperatively, the patient developed neck pain with limited mobility. Treatment included oral administration of Eperisone Hydrochloride tablets 50 mg three times daily and Sodium Aescinate tablets 30 mg twice daily. The patient discontinued these oral medications after 7 days and did not obtain further supplies.

The patient presented to our Tuina department with unresolved neck pain despite post-discharge oral medication. He reported persistent rightward neck deviation, pain, and restricted movement beginning two weeks after tonsillectomy and adenoidectomy, which showed no improvement with rest or medication. Current symptoms included significant left-sided neck pain, rightward head tilt, limited cervical mobility, and occasional headache and dizziness, without limb numbness or other discomfort.

### 2.2 Physical examination

Physical examination revealed cervical muscle rigidity, tenderness over the left atlantoaxial joint and marked tenderness along the left paravertebral regions from the third cervical vertebra (C3) to the fifth cervical vertebra (C5). Cervical motion was significantly limited in extension, flexion, and left rotation, with mild restriction in right rotation. Patient symptoms were assessed using the Short-form McGill Pain Questionnaire (SF-MPQ), which incorporates the Visual Analogue Scale (VAS), Present Pain Intensity (PPI), and Pain Rating Index (PRI), along with the Neck Disability Index (NDI). The evaluation revealed scores of 7 on the VAS, 4 on the PPI, and 10 on the PRI, resulting in a total SF-MPQ score of 21. The NDI score was 31. These findings indicate significant neck pain and functional impairment in the patient.

### 2.3 Imaging assessment

**Figure 1:**
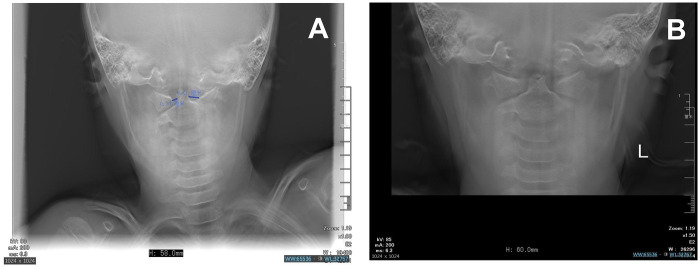
Pre-treatment and post-treatment DTS radiographic features. **(A)** Pre-treatment DTS revealed asymmetric widening of the distance between the odontoid process axis and the lateral masses. The left lateral mass interval was significantly widened to approximately 0.6 cm, while the right interval measured approximately 0.3 cm. A steplike change was observed at the lateral margins of the atlantoaxial vertebral bodies. These imaging features are consistent with atlantoaxial subluxation. **(B)** Post-treatment DTS revealed normal alignment and intact bone architecture without evidence of fracture. The atlantodental intervals appear slightly asymmetric, with mild widening noted on the left side. No significant displacement of the lateral masses is observed.

Digital tomosynthesis (DTS) of atlantoaxial joint indicated findings consistent with atlantoaxial subluxation (Figure 1A), and cervical digital radiography (DR) demonstrated reversal of the physiological curvature.

### 2.4 Diagnosis

Based on the clinical history, symptoms, and imaging results, a diagnosis of GS (non-traumatic atlantoaxial subluxation after tonsillectomy and adenoidectomy) was established.

## 3 Treatment and intervention

The patient had previously been managed with oral pharmacotherapy. However, this treatment did not result in significant pain relief. During the current treatment period, the patient was not instructed to continue taking any oral medications. Furthermore, the patient declined both cervical traction and surgical intervention. Given these circumstances, manipulative therapy (Tuina) was considered a viable option. This approach is non-invasive, relatively safe, and potentially effective. The initial treatment plan consisted of manual stimulation applied to the tonsillar reflex zone, followed by a four-step manipulative reduction. The patient received a total of 9 days of treatment. During the first three days, manual stimulation of the tonsillar reflex zone and tendon-regulation Tuina manipulation were performed once daily, with each session lasting approximately 20 min. On days 4 through 9 of the treatment period, the above stimulation and manipulation were applied first at the same frequency and duration, followed by the four-step manipulative reduction once daily for about 5 min. The total duration of each treatment session was 25 to 30 min. All manipulations described in this report were performed by a single licensed Tuina therapist with more than 10 years of clinical experience, including specific training in atlantoaxial manipulative techniques. The treatment timeline is presented in Supplementary Figure 1. The specific treatment steps were as follows.

### 3.1 Manual stimulation of the tonsillar reflex zone

**Figure 2:**
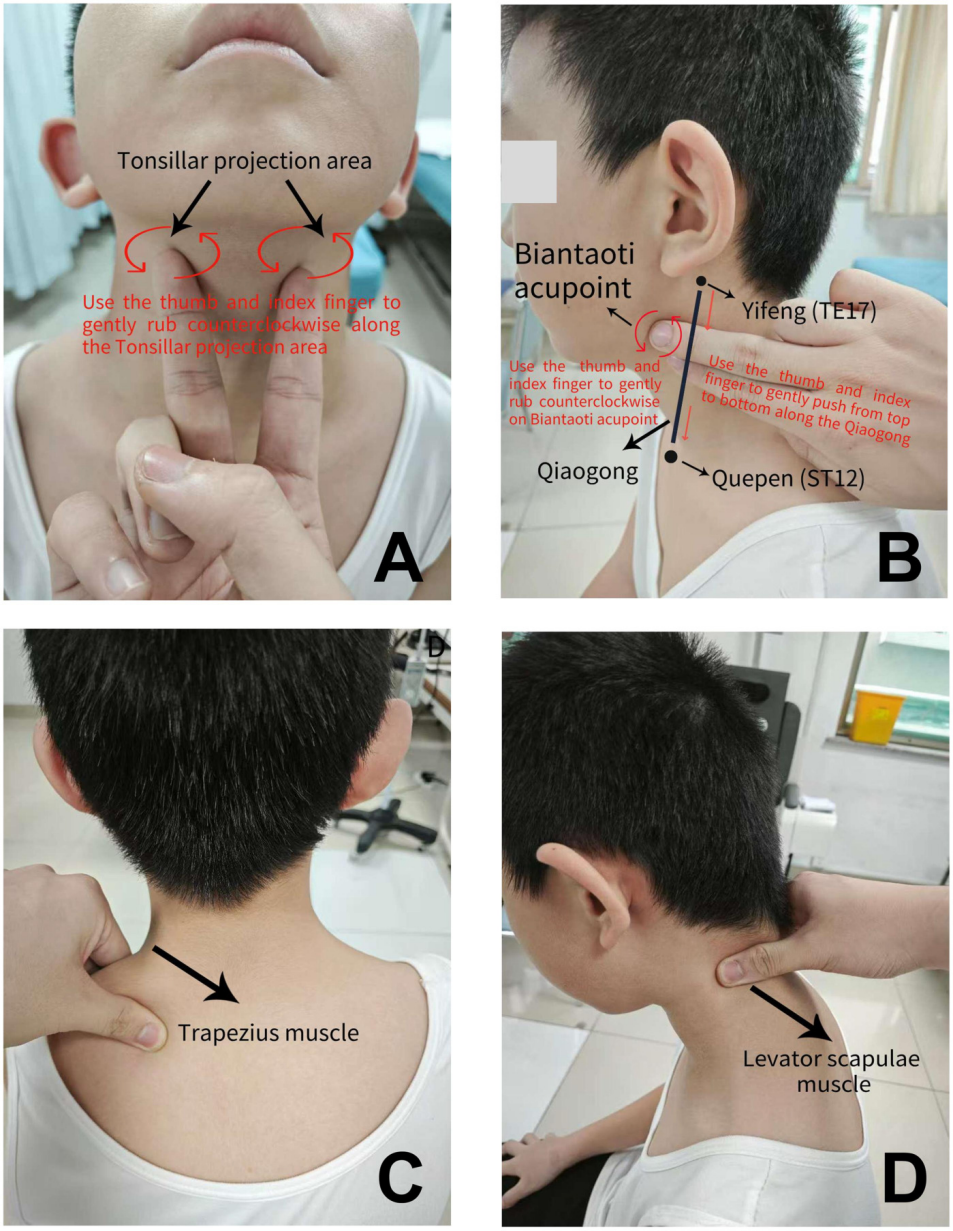
Manual stimulation of the tonsillar reflex zone and tendon-regulation tuina. **(A)** Applied gentle hooking and kneading manipulations on the surface tonsillar projection area. **(B)** Gently stroke downward along the Qiaogong region and applied hooking and kneading manipulations on the Biantaoti acupoint. **(C)** Tendon-regulation Tuina is applied to the trapezius muscle. **(D)** Tendon-regulation Tuina is applied to the levator scapulae muscle.

All stimulation maneuvers on the tonsillar reflex zone were performed externally. The patient was positioned sitting with the neck slightly extended to expose the bilateral Qiaogong region. This region is defined as the area along the line from Yifeng (TE17) to Quepen (ST12), corresponding to the sternocleidomastoid muscle zone extending from the sternal end of the clavicle to the mastoid process. The therapist stood slightly behind and to the side of the patient. Using the thumb, index, and middle fingers, the affected tonsillar reflex zone was grasped and kneaded. A manual pressure of approximately 3 to 5 Newtons was applied, gradually increasing from light to moderate intensity as tolerated by the patient. This kneading was sustained for about 3 min. Prior to the procedure, a small amount of medical Vaseline was applied to the area to reduce friction and prevent skin irritation. Subsequently, using the index and middle fingers, the therapist performed a gentle, soothing pushing motion downward along the tonsillar reflex zone for 4 min at a frequency of 60 times per minute, always prioritizing patient comfort. Finally, the therapist placed the index and middle fingers on the cutaneous projection area of the tonsils and on the Biantaoti acupoint (located 3.5 cm directly below the mandibular angle) to perform gentle hooking, pinching, and kneading, repeating this motion 100 to 300 times (Figures 2A,B).

Subsequently, tendon-regulation Tuina manipulation was performed. The therapist applied techniques including grasping the five meridians, pressing and kneading, and one-finger meditation pushing to the meridians of the patient's head and neck. The manipulation pathway proceeded from the Governor Vessel to the Foot-Shaoyang Gallbladder Meridian, moving from medial to lateral and anterior to posterior. Concurrently, in accordance with the distribution characteristics of the different muscles in the head and neck region, the depth of manipulation was progressively increased from superficial to deep layers. The treatment area extended from the forehead to the posterior neck, with manipulations applied along the direction of the muscle fibers. The primary target areas were the suboccipital triangle muscles, the trapezius, and the levator scapulae (Figures 2C,D). The force applied was adjusted to a level tolerable for the patient. These Tuina manipulations continued for approximately ten minutes.

### 3.2 Four-step manipulative reduction

Three days later, the patient returned to continue treatment. He reported reduced pain, decreased muscle stiffness, and slight improvement in cervical range of motion. A manipulative reduction procedure was initiated on the same day. The specific protocol involved muscle relaxation through tendon-regulation manipulation, followed by a four-step manipulative reduction.

**Figure 3:**
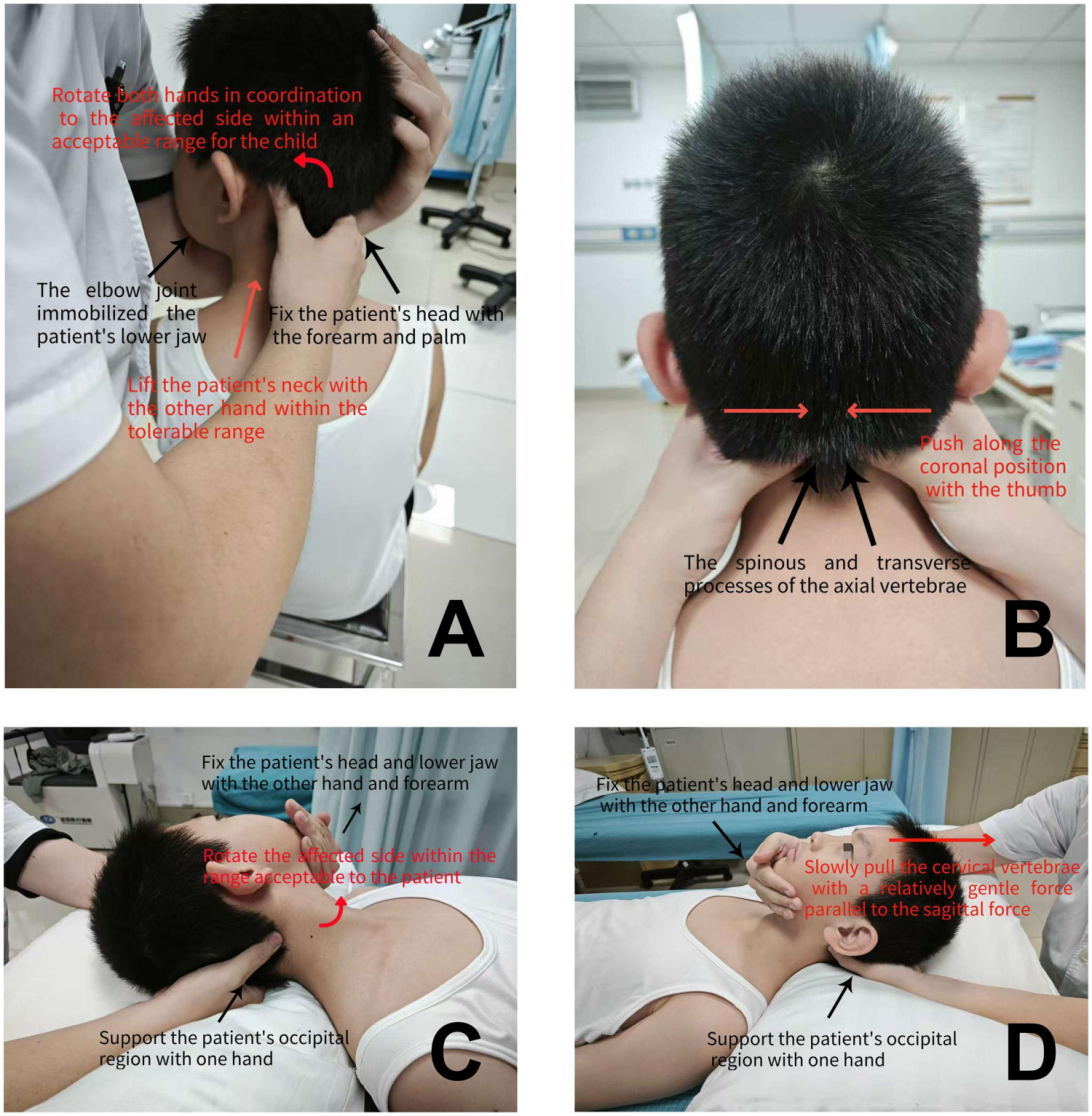
Four-step manipulative reduction.

First, the patient is instructed to maintain head anteflexion at an angle of 15–30°. The therapist stands behind the patient, securing the mandible with the elbow while stabilizing the head with the palm and forearm. Within the patient's tolerance, a rotational lifting maneuver is performed toward the affected side, with the rotation angle controlled between 10 and 15° and the force applied adjusted to avoid significant discomfort (Figure 3A). Subsequently, based on the degree of atlantoaxial subluxation identified in the first step, the therapist uses the thumb of the opposite hand to apply a force parallel to the coronal plane against the spinous process of the axis on the affected side. The initial force is approximately 0.5–1 kg and is gradually increased to the maximum level tolerable by the patient, not exceeding 3 kg (Figure 3B). Next, the patient is positioned supine. The therapist supports the occiput with one hand and stabilizes the mandible and head with the other, gently rotating the head toward the affected side within a range of 15–20°, as tolerated by the patient (Figure 3C). Finally, with the patient still supine, the therapist supports the occiput with one hand and stabilizes the mandible and head with the other, applying gentle traction along the sagittal plane. The traction force maintained at 2–5 kg for about 1–2 min (Figure 3D). The entire procedure lasted approximately 5 min. A slight gliding sensation under the fingers was considered appropriate during manipulation, and any pursuit of audible cracking was strictly avoided. Meanwhile, the rotation angle during cervical manipulation should be controlled within 20°. The patient should be promptly asked whether pain occurs. If any discomfort arises, treatment should be terminated immediately.

## 4 Safety assessment

During and after treatment, patients should be closely monitored for neurological complications such as limb numbness, weakness, sensory abnormalities, or gait instability. The risk of vertebral artery injury should also be assessed. Furthermore, children are still in a stage of growth and development. The stability of their atlantoaxial joint primarily depends on the transverse ligament, alar ligaments, and dentate ligaments. Unlike adults, children have incompletely developed atlantoaxial structures, smaller joint components, and predominantly cartilaginous bone tissue, resulting in poorer stability and a greater susceptibility to atlantoaxial imbalance. Consequently, they face a higher risk of atlantoaxial subluxation. Before performing manipulative therapy, a comprehensive evaluation of the child's atlantoaxial condition is necessary. It is essential to rule out congenital structural anomalies of the atlantoaxial joint, the presence of congenital tumors in the neck or cranium, and congenital instability caused by ligamentous abnormalities such as rupture or hypoplasia of the transverse ligament, alar ligaments, or ligamentum flavum. In this case, DTS examination revealed well-developed anterior and posterior arches of the atlas, a normal odontoid process morphology without hypoplasia or bony abnormalities, and continuous occipital and cervical bone structures without signs of destruction or pathological fracture. These findings largely ruled out congenital structural anomalies of the atlantoaxial joint and cervical spine as well as primary bone tumors. The patient had undergone tonsillectomy and adenoidectomy at our hospital before symptom onset, with a rigorous perioperative evaluation including nasopharyngeal and cranial CT scans that showed no abnormal densities or space-occupying lesions, thereby essentially excluding nasopharyngeal or intracranial neoplasms. History taking indicated that prior to disease onset, the patient had no symptoms such as neck pain or restricted mobility, and the cervical rotation range fell within the normal range for children. This essentially ruled out atlantoaxial instability due to ligamentous rupture or laxity. Taken together, the imaging and physical examination findings confirmed the absence of congenital structural abnormalities, providing a safe foundation for performing manipulative therapy. A comprehensive neurological examination and cervical spine function assessment were conducted before and after treatment to ensure therapeutic safety.

## 5 Treatment outcomes

**Figure 4:**
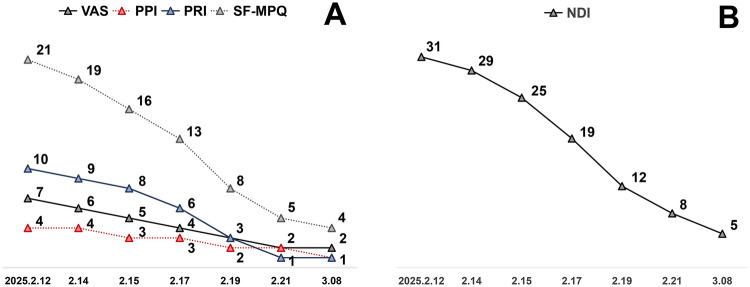
Longitudinal changes in assessment indicators during the treatment course. **(A)** Changes in SF-MPQ scores. The SF-MPQ includes VAS, PPI, and PRI. **(B)** Changes in NDI scores.

Following a 9-day treatment course, the patient returned for reassessment. He reported improved mobility of the neck. Physical examination revealed that the neck had regained a natural, vertical alignment. The patient was able to achieve bilateral rotation of the cervical spine, although a slight residual limitation remained during left rotation. Palpation indicated that tenderness over the left atlantoaxial joint and the paravertebral soft tissues at C3/4 and C4/5 was reduced. DTS showed that the left atlantoaxial joint space had widened compared to earlier findings (Figure 1B). Follow-up assessment with SF-MPQ and NDI demonstrated significant improvement. The VAS score was 2, the PPI score was 2, the PRI score was 1, with the total SF-MPQ score was 5 (Figure 4A). The NDI score decreased to 5 (Figure 4B). These findings indicated a reduction in both the patient's pain level and functional impairment. Based on these clinical and imaging improvements, the patient's symptoms of Grisel syndrome were substantially resolved. At the 15-day follow-up, the therapeutic effect of tonsillar reflex zone stimulation combined with the four-step manipulative reduction remained continuous, with no recurrence of clinical symptoms.

## 6 Discussion

### 6.1 Diagnostic basis of GS

Currently, no universally established diagnostic criteria exist for GS. However, it is widely recommended that GS should be considered and suspected in patients presenting with neck pain or torticollis following otorhinolaryngological surgery (3). Diagnosis primarily relies on clinical signs and imaging findings. Common symptoms include torticollis, neck pain and stiffness, and restricted neck movement. A study reported that patients with GS exhibit marked elevation of the ipsilateral trapezius muscle, which helps distinguish it from cervical dystonia where the contralateral trapezius is typically elevated (4). Additionally, patients often cannot rotate the neck, even transiently, and significant contraction of the ipsilateral sternocleidomastoid muscle can be observed during attempted rotation. This finding may serve as an indicative sign for GS. On physical examination, increased muscle tone in the neck, tenderness over the occipital area, upper cervical paraspinal regions, or sternocleidomastoid insertion points may be present. Some patients may also show neurological changes, ranging from mild symptoms such as upper limb numbness and pain due to cervical nerve root compression, to severe manifestations like limb weakness and gait instability resulting from spinal cord compression.

### 6.2 Pathophysiological mechanisms of GS

The pathogenesis of GS remains incompletely understood. Battiata first proposed the two-hit hypothesis, suggesting that two conditions are required for its development. First, pre-existing laxity of the C1-C2 ligaments (e.g., the transverse ligament), often manifested as an increased atlantodental interval. And second, the spread of inflammatory mediators via the pharyngovertebral venous plexus to the cervical muscles, causing spasm and subluxation (5). Based on this hypothesis, the currently accepted mechanism involves an initial trigger, typically a head and neck infection (e.g., suppurative tonsillitis, retropharyngeal abscess), that induces local inflammation affecting the periarticular ligaments of the atlantoaxial joint, leading to ligamentous edema and laxity. Inflammatory cytokines such as interleukin (IL) -6 and tumor necrosis factor-α (TNF-α) may directly damage collagen fibers in the ligaments, further reducing joint stability. Secondary injury factors also contribute, due to weaker neck muscles and more lax joint capsules in children, actions such as coughing, vomiting, sneezing, or sudden neck movements can exert abnormal shear forces on the already compromised atlantoaxial joint. The interplay of these pathological factors ultimately leads to the onset of GS. Tonsillectomy and adenoidectomy, as primary treatments for tonsillar and adenoid diseases, are frequently associated with postoperative GS. Follow-up data from one hospital identified 17 cases of atlantoaxial subluxation following adenotonsillectomy between January 2020 and December 2024 (6).

Based on the pathogenesis hypothesis of GS, the reasons for its occurrence after adenotonsillectomy can be summarized as follows. First, patients often have a history of recurrent acute tonsillitis. The tonsils act as a potential infectious focus, and surgical trauma and stress can reactivate these latent foci. Inflammatory swelling in the open surgical site may indirectly compress and stretch peri-cervical ligaments. Second, the ligaments in the C1-C2 region are generally more lax in children, predisposing them to subluxation. Although no direct evidence confirms that tonsillar and adenoid hypertrophy alters cervical ligament anatomy, a study indicated that pharyngitis following COVID-19 infection could lead to laxity in the ligaments around the atlantoaxial joint (7). It is thus plausible that upper respiratory infections causing tonsillar and adenoid hypertrophy may contribute to atlantoaxial ligament involvement and GS. Third, patients undergoing adenotonsillectomy are often placed in the Robin position. This position requires the patient to lie supine with the head elevated. Under general anesthesia and muscle relaxants, neck muscles become further relaxed, and excessive neck extension combined with inherent ligamentous laxity may compromise atlantoaxial stability in children. Fourth, improper surgical technique, such as excessively deep resection injuring the prevertebral fascia or localized infection spread, can cause enlargement of retropharyngeal or prevertebral lymph nodes. Enlarged lymph nodes may traction the ligaments, leading to restricted atlantoaxial motion or subluxation (8). Finally, patients remain under general anesthesia post-surgery, and rough handling during transfer may also predispose to atlantoaxial subluxation if nursing care is not carefully executed.

### 6.3 Imaging evidence of GS

Imaging examination represents one of the most valuable tools for confirming GS. Currently, open-mouth and lateral cervical radiographs remain the primary imaging modalities for diagnosing atlantoaxial subluxation in clinical practice. DR of the open-mouth view can reveal asymmetry in the distance between the odontoid process and the lateral masses of the atlas, inconsistency in the atlantoaxial lateral mass joint spaces, and misalignment of the upper cervical lateral masses. The lateral cervical view demonstrates the distance between the odontoid process and the anterior arch of the atlas (9). However, obtaining these views requires patient cooperation in positioning, which may be limited by pain, potentially leading to incomplete examinations. Some researchers consider three-dimensional reconstructed cranial cervical CT to be the optimal imaging modality for diagnosing atlantoaxial subluxation. It clearly reveals the extent and type of subluxation without interference from patient positioning or image overlap, and enables multi-planar visualization of the atlantoaxial joint through post-processing. Furthermore, this technique is essential for evaluating the degree of atlantoaxial displacement, playing a key role in determining the severity of subluxation and guiding appropriate treatment strategies.

The Fielding and Hawkins grading system is commonly used in clinical practice to evaluate atlantoaxial subluxation. This system categorizes patients based on craniocervical three-dimensional CT findings (10). Type Ⅰ is characterized by rotational fixation with anterior atlantoaxial displacement of ≤3 mm. Type Ⅱ involves rotational fixation with anterior displacement between 3 and 5 mm. Type Ⅲ refers to rotational fixation with anterior displacement exceeding 5 mm. Type Ⅳ primarily includes rotational fixation accompanied by posterior atlantoaxial displacement. However, craniocervical three-dimensional CT has limited capacity for soft tissue assessment. It can only infer ligamentous and soft tissue injury indirectly by measuring indicators such as the atlantodental interval and the difference in the distance between the dens and lateral masses bilaterally (11). Additionally, during this examination, patients are required to remain in a supine position, the radiation exposure for children is relatively high, and the procedure is time-consuming.

A study suggested that magnetic resonance imaging (MRI) provides a comprehensive evaluation of paravertebral soft tissues, joints, and ligaments. Moreover, it can demonstrate evidence of vertebral distraction and spinal cord compression, making it suitable as a primary diagnostic imaging modality for GS. However, they also noted that some children have difficulty tolerating the confined space and noise during MRI, limiting its widespread clinical use (12). In recent years, x-ray DTS has gained considerable attention for diagnosing atlantoaxial subluxation. A study compared DR and DTS in patients with suspected atlantoaxial subluxation: among 43 cases examined with DR, 20 (46.50%) were diagnosed with subluxation, whereas among 17 cases examined with DTS, 15 (88.20%) were diagnosed, indicating a significantly higher diagnostic rate with DTS (13). Another study noted that DTS reduces tissue overlap compared to DR, allowing clearer visualization of complex anatomical structures such as the atlantoaxial joint (14). Compared to CT, DTS reduces radiation exposure and improves in-plane resolution. Additionally, DTS does not require an open-mouth position for atlantoaxial imaging. Continuous, multi-layered coronal or sagittal images of the atlantoaxial region can be obtained through one scan and image reconstruction. The equipment space is open and the patient's willingness to cooperate is high (13). DTS overcomes the limitations of DR, CT, and MRI. However, as a lower-resolution tomographic technique, it fails to fully distinguish soft tissue structures, cannot assess the impact of ligamentous injury on atlantoaxial stability, and does not determine the severity of atlantoaxial subluxation. Additionally, DTS lacks information on the rotational angle of the atlantoaxial joint and is unable to diagnose specific types of atlantoaxial subluxation. In summary, DTS offers unique advantages and considerable promise in the diagnosis of atlantoaxial dislocation, though further investigation is warranted to establish its precise clinical role.

### 6.4 Current therapeutic approaches to GS

Clinical treatment strategies for atlantoaxial subluxation are largely based on the Fielding and Hawkins grading system (15). For patients with type Ⅰ subluxation and no neurological deficits, recommended treatment includes antibiotics, anti-inflammatory medications, muscle relaxants, and cervical immobilization. In cases of fixed subluxation or the presence of neurological symptoms, invasive management using halo vest immobilization may be considered. After successful reduction, patients are required to maintain cervical collar fixation for at least three months. In the most severe cases involving recurrent subluxation or difficult reduction, atlantoaxial fusion is clinically valuable. In some instances, C0-C1-C2 arthrodesis may be necessary (16).

The management of GS should address two aspects. First, it is essential to alleviate inflammatory swelling in the open surgical site after tonsillectomy, thereby reducing indirect compression and ligament traction on surrounding cervical tissues. Second, reduction of the displaced atlantoaxial joint is necessary to relieve soft tissue injury and potential nerve compression. The Qiaogong region corresponds to the sternocleidomastoid muscle zone. Deep to this area lies the carotid sheath, a fascial structure enveloping the common carotid artery, internal carotid artery, internal jugular vein, and vagus nerve. The external carotid artery, a terminal branch of the common carotid, supplies the tonsils via branches such as the facial artery, ascending pharyngeal artery, and maxillary artery. Stroking the Qiaogong region applies mechanical stimulation that dilates the common carotid artery, increases blood flow, and enhances perfusion through the external carotid and its branches. This promotes postoperative wound healing, angiogenesis, and blood supply restoration in the surgical area. A study observed significantly elevated IL-1β expression during inflammatory episodes in the tonsillar region (17). Multiple studies have confirmed increased serum IL-6 levels during tonsillar diseases, often accompanied by lymphocytopenia (18). Some reports also noted distinct microbial communities in inflamed tonsils compared to healthy controls, suggesting that tonsillectomy may disrupt the relatively stable microbiota and provoke postoperative inflammation (19).

As a traditional external therapy, Tuina has demonstrated anti-inflammatory effects. Some researchers observed that local Tuina application in rat models of knee osteoarthritis significantly alleviated pain and swelling while reducing serum levels of IL-1β, IL-17, matrix metalloproteinases (MMP) -3, and MMP-13 (20). A study demonstrated that pediatric Tuina therapy reduced inflammatory responses and prostaglandin E2 (PGE2) expression in the blood and hypothalamus of lipopolysaccharide (LPS)-induced febrile young rabbits. Plasma metabolomics analysis revealed that during the antipyretic and anti-inflammatory processes induced by Tuina therapy, multiple potential biomarkers were identified. These primarily included riboflavin, nicotinamide N-oxide, porphyrinogen, 5-hydroxyindoleacetic acid, gamma-aminobutyric acid, and lysophosphatidylcholine (21). In addition, the Biantaoti acupoint has been validated by clinicians for its efficacy in treating tonsillitis, pharyngitis, parotitis, and hoarseness through Tuina manipulation. Notably, although we have proposed a potential anti-inflammatory mechanism based on evidence from animal studies and other clinical conditions including the modulation of inflammatory cytokines, no serum or local inflammatory markers were measured in this case report. Therefore, the proposed anti-inflammatory effect of stimulating the tonsillar reflex zone remains speculative in the context of this patient.

As a preoperative preparation for the four-step manipulative reduction, tendon-regulation Tuina manipulation is considered in modern medical research to exert dual effects. On the one hand, it generates thermal effects that accelerate blood circulation in the head and neck region, thereby enhancing muscular contractility and elasticity (22). On the other hand, the manual techniques stimulate tendon spindles to intensify local sensory input, activating efferent fibers and muscle stretch receptors while increasing receptor sensitivity. This process indirectly stimulates the sensory cortex and inhibits the brainstem reticular formation, ultimately reducing local muscle tension and relieving spasms. These effects collectively diminish resistance from rigid muscles during subsequent reduction maneuvers. The four-step manipulative reduction begins with positioning the patient's head in a forward-inclined posture. Utilizing principles of traction, leverage, and rotation, a controlled rotatory lifting force is applied within the patient's tolerable range. The second step involves syndrome-differentiated manipulation based on anatomical alterations in the atlantoaxial joint: graded forces parallel to the coronal plane are applied to push the transverse and spinous processes of the axis, facilitating restoration of normal anatomical alignment. In the third step, rotational maneuvers in different positions readjust the atlantoaxial joint from alternative angles, enhancing reduction efficacy. The final step employs sagittal traction to alleviate transient discomfort from preceding manipulations, simultaneously widening intervertebral spaces and reducing articular pressure, thereby enabling atlantoaxial realignment in a more relaxed mechanical environment. The four-step manipulative reduction maneuver described above is non-invasive. Patients treated with this manipulation have shown varying degrees of clinical improvement and corresponding radiographic changes. Importantly, no significant restriction of cervical spine function has been observed following treatment in multiple clinical applications. However, this technique requires execution by a trained therapist, as improper performance may pose a risk of secondary injury. While this approach offers a potential therapeutic option for various types of atlantoaxial subluxation, its efficacy and safety warrant further validation through rigorous clinical studies.

### 6.5 Limitations and deficiencies of the study

This case report has certain limitations. The diagnosis of GS in this patient was established through atlantoaxial DTS examination, combined with clinical symptoms and the clinician's experience. However, due to considerations regarding the patient's economic status and treatment compliance, craniocervical three-dimensional CT and cervical MRI were not performed during this diagnostic process. Consequently, we were unable to obtain information regarding ligamentous injuries in the cervical region or determine the Fielding classification. This limitation made it difficult to accurately assess the severity of the atlantoaxial subluxation and precluded the possibility of excluding special types of atlantoaxial subluxation, thereby increasing the diagnostic and therapeutic risk to some extent. This also suggests that if conditions permit, patients should undergo a complete imaging examination to better guide clinical diagnosis and treatment.

Furthermore, the therapeutic approach employed in this case cannot be fully quantified. During the manipulation procedures, the force applied was primarily adjusted based on the therapist's clinical experience and the patient's tolerance level. No objective quantification tools such as handheld dynamometers or goniometers were used in this situation. Future studies should consider integrating such devices to improve reproducibility. Meanwhile, the quantification requirements of this therapeutic approach still need to be explored and validated through high-quality, large-scale clinical trials. Uniform training standards should also be established for all therapists to ensure trial quality. In addition, the post-treatment follow-up duration in this case was relatively short. Although the patient demonstrated symptomatic improvement at both the immediate post-treatment assessment and the 15-day follow-up, with no evidence of symptom recurrence, the durability of this therapeutic effect beyond two weeks remains unclear. Longer-term follow-up is necessary to determine whether the observed benefits are sustained over months or years. We acknowledge this limitation and recommend that future prospective studies or case series include extended follow-up periods to confirm the long-term durability and safety of this combined manipulative approach for pediatric GS.

## 7 Conclusion

In summary, this case report aimed to explore a therapeutic approach for GS following tonsillectomy and adenoidectomy. Manual stimulation of the tonsillar region was performed to alleviate local inflammation in this area. Subsequently, Tuina relaxation therapy was applied to the cervical muscles and soft tissues, followed by a four-step manipulative reduction to correct the positional relationship of the atlantoaxial joint, thereby improving the clinical symptoms in this patient. As this report is based on the observation and analysis of a single case, further randomized controlled trials are required in future clinical applications to validate the efficacy and safety of this treatment protocol. Nevertheless, this combined therapy, as a traditional external treatment technique characterized by non-invasive, wide applicability, and a long-standing history, may be more readily accepted by pediatric patients and their parents. Therefore, it holds promise for providing additional treatment options for children with GS.

## Data Availability

The original contributions presented in the study are included in the article/Supplementary Material, further inquiries can be directed to the corresponding author.
