# Supplementary figures and images for: Combined tonsillar reflex zone stimulation and four-step manipulative reduction for Grisel syndrome following pediatric adenotonsillectomy: a case report
Source: Front Pediatr. 2026 Jun 3;14:1744987. doi: 10.3389/fped.2026.1744987 (PMC13272336; doi:10.3389/fped.2026.1744987)

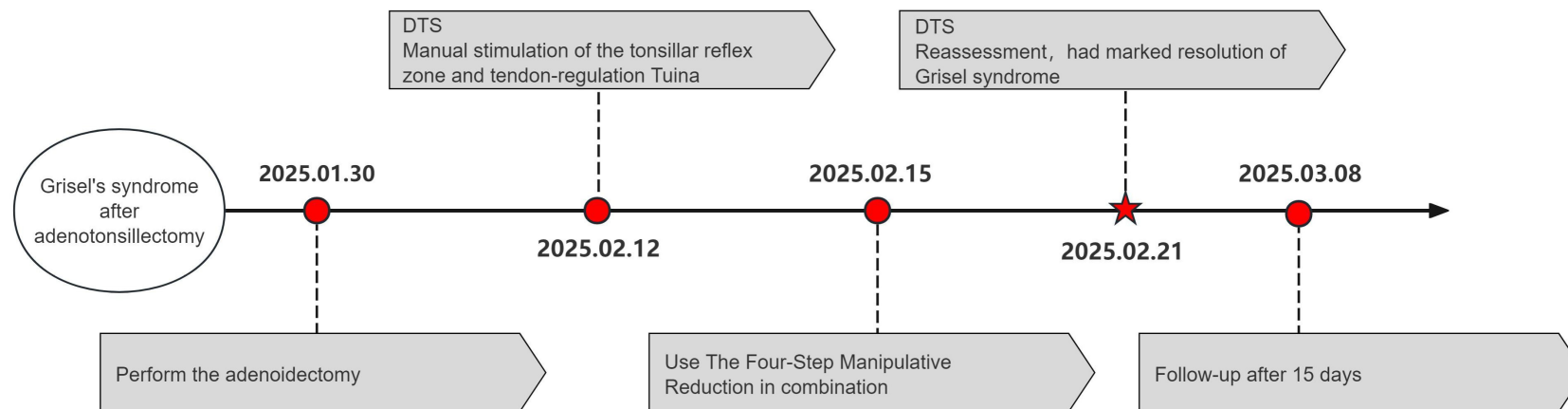

**Supplementary Figure 1.** The specific timeline of this treatment.

Supplement: Supplementary file 1 [file image1.pdf]
